# Conceptualization, development, and early dissemination of eMPACT^TM^: A competency-based career navigation system for translational research professionals

**DOI:** 10.1017/cts.2023.693

**Published:** 2023-12-11

**Authors:** Ikseon Choi, Sejung Kwon, Jay W. Rojewski, Janette R. Hill, Eunice S. Kim, Elaine Fisher, Rebecca S. Thomas, Linda McCauley

**Affiliations:** 1 Nell Hodgson Woodruff School of Nursing, Emory University, Atlanta, GA, USA; 2 Department of Workforce Education and Instructional Technology, University of Georgia, Athens, GA, USA; 3 CenExel: Medical Research Centers, Atlanta, GA, USA

**Keywords:** Translational workforce development, clinical research coordinator, career navigation system, job and training matching algorithm, career advancement

## Abstract

**Introduction:**

Purposeful training and ongoing career support are necessary to meet the evolving and expanding roles of clinical research professionals (CRP). To address the training and employment needs of clinical research coordinators (CRCs), one of the largest sectors of the CRP workforce, we designed, developed, and implemented an online career navigation system, eMPACT^TM^ (eMpowering Purposeful Advancement of Careers and Training).

**Methods:**

A design-based research method was employed as an overarching approach that frames iterative design, development, and implementation of educational interventions. The five major phases of this project – conceptualization, task analysis for measurement development, algorithms development, algorithms validation, and system evaluation – presented specific goals and relevant methods.

**Results:**

The results reported how the eMPACT^TM^ system was conceptualized, developed, and validated. The system allowed CRCs to navigate tailored training and job opportunities by completing their task competencies and career goals. The data sets could, in turn, support employees’ and training coordinators’ informed decisions about organizational training needs and recruitment. The early dissemination results showed steady growth in registered CRCs and diversity in users’ ethnicity and job levels.

**Conclusions:**

The eMPACT^TM^ service showed the possibility of supporting CRCs’ individual career advancement and organizational workforce enhancement and diversity. Long-term research is needed to evaluate its impact on CRC workforce development, explore key factors influencing workforce sustainability, and expand eMPACT^TM^ service to other CRP sectors.

The roles of clinical research professionals continue to evolve and expand as the number and complexity of research projects increase. Clinical research coordinators (CRCs) are critical to project success, and their roles and responsibilities often vary from project to project, including day-to-day administrative tasks, clinical trial operations, acting as liaisons between principal investigators and subjects, specimen collection, data management, and reporting [1–4]. Ongoing training is necessary to meet the increasing demands placed on health professionals when conducting research and to maintain a highly qualified workforce.

Job stress is one of the key contributors to a high turnover rate among CRCs [5,6]. Staff turnover is not only expensive but threatens the timely and successful completion of clinical research projects [7]. Factors contributing to job stress and burnout include gaps in training and mentoring, particularly a lack of purposeful training opportunities and connections with experienced CRCs offering advice to advance one’s career [8–10]. Creating workforce support mechanisms that promote career advancement, job satisfaction, and professional well-being is critical.

The prevailing training paradigm in translational sciences usually reflects an organization-based, top-down perspective where responsibility for ensuring a competent clinical workforce is assumed by organizational representatives (e.g., training coordinators and human resources [HR] personnel). Institutionally driven training efforts are designed to meet regulatory standards, maintain organizational safety, mitigate risk, and establish performance standards [11]. From this perspective, training typically overlooks individual employee career needs or goals, particularly when the job is time-limited by funding. Consequently, most translational workforce development efforts for clinical research professionals address institutional training and are evaluated by the degree to which organizational performance is maximized.

Our goal was to develop a practical approach to workforce training that satisfied organizational needs for a competent workforce while simultaneously supporting employees’ career goals related to advancement as clinical research professionals. One challenge we faced was providing opportunities for employees to exercise personal agency in determining need or desired workforce development and training activities. In this context, we developed an online multi-faceted career navigation system, eMPACT^TM^ (eMpowering Purposeful Advancement of Careers and Training), to address CRCs’ training and employment needs by supporting their self-directed professional development. Users of the eMPACT^TM^ system can apply knowledge of self by identifying personal workstyle attributes [12], CRC task competencies, and their career goals toward making informed decisions about organizational training needs and availability, as well as employment requirements and opportunities. The conceptual framework of eMPACT^TM^, the development of core system functions, the validation of system algorithms, and the results of initial dissemination are presented.

## Method

A design-based research (DBR) method was employed as our overarching approach that frames iterative design, development, and implementation of educational interventions to advance both theories and practices [13–15]. DBR, as an educational research method, is highly aligned with translational science that focuses on translating basic biomedical science to healthcare practices for societal impact. Thus, the current project can also be characterized as “translational education” research [16]. The project was laid out in progressive phases of development and evaluation. The five major phases of this project were conceptualization, task analysis for measurement development, algorithms development, algorithms validation, and system evaluation. Each phase was defined by specific goals and relevant methods to yield intended results (see Table 1).

**Table 1.** Translational phases and their methodological approaches

Phase	Translational task	Goals	Data sources	Expected results
1	Conceptualization of eMPACT^TM^ system	Develop framework to orient needs, goals, and system functions	Literature review; Prior needs assessment	Systemic conceptual framework; System function specifications
2	Task analysis for measurement development	Develop measure of core competencies	Literature review; Expert interviews	Competency survey
3	Algorithms development	Develop matching algorithms using competency survey	Literature review	Profile matching algorithms
4	Algorithms validation	Validate functions of algorithems	Hypothetical simulation	Validation of algorithms, functions, user experiences
5	System evaluation through early dissemination	Preliminary evaluation of dissemination trends in diversity	User growth; system access; demographics	Detect early signals to predict the dissemination trends

## Results

### Phase 1: Conceptualization of Career Advancement Cycle through Purposeful Training

**Figure 1.**
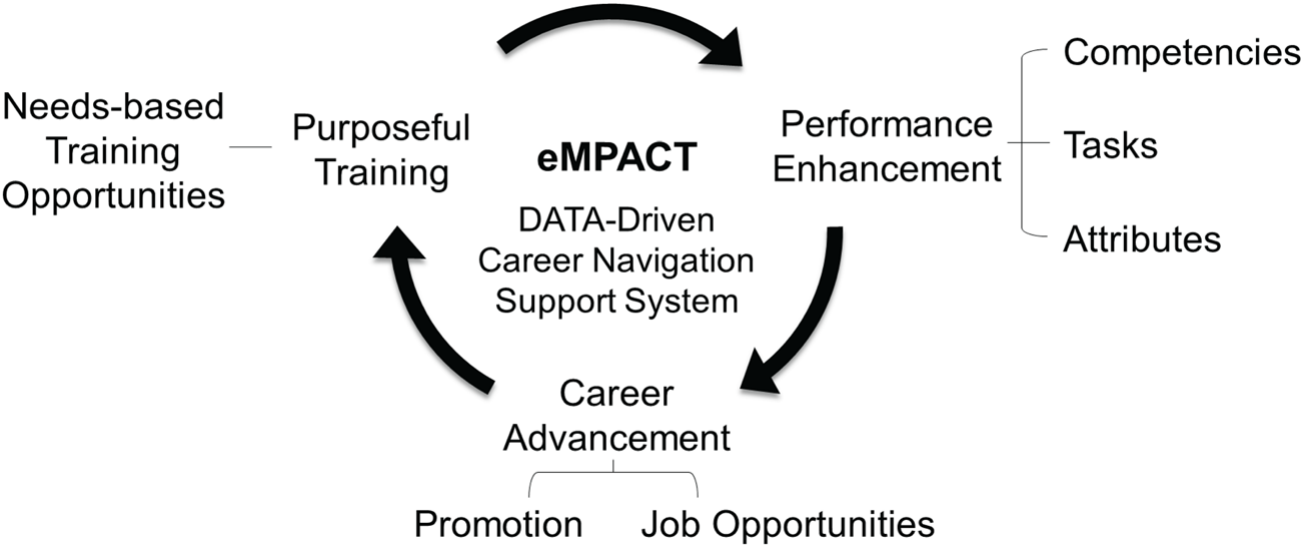
Purposeful training and career advancement cycle promoted by eMPACT^TM^.

The first step in developing the eMPACT^TM^ system was determining the desired outcomes for the program and the possible impacts of the proposed solutions. This section explains our desired outcome – a positive and sustainable career advancement cycle that benefits both employees and organizations (see Fig. 1).

#### Purposeful training

Training is the most common means of supporting employees’ career advancement [17] by enhancing employees’ knowledge and skills to perform current or future job duties [18]. Effective training is purposeful – developed to address specific organizational needs while being informed by individual workers’ needs. Thus, expected training results improve work performance, benefiting employees and the organization (see Fig. 1).

#### Performance enhancement

Job performance depends on an individual’s competencies, types of tasks, and the social and physical environments encountered in the workplace [19]. Competencies refer to an individual’s core capability to perform specific tasks [20]. Job descriptions identify expected duties and related job tasks that can be translated into competencies (e.g., skills and knowledge) needed to perform the tasks. Individuals’ workstyle attributes (e.g., achievement orientation, social influence, interpersonal orientation, adjustment, conscientiousness, independence, and practical intelligence) are important mediating factors in determining performance outcomes [21]. Thus, identifying employee competencies and workstyle attributes that can be aligned with required job tasks is vital for understanding and predicting performance outcomes for the job of CRC.

#### Career advancement

Career advancement is the long-term process of discovering and achieving one’s career goals by accumulating work experience, acquiring advanced training, and identifying and pursuing future positions [22]. Career advancement reflects an individual’s personal and professional goals and the growth accrued to meet these goals. Collectively, career advancement can be a critical indicator of current and emerging trends for workforce progression, representing an ongoing negotiation of individuals’ career goals, organizational goals, and societal needs. How, then, can career information be matched to facilitate informed career-related decisions and behaviors?

#### Empowerment through data-driven decision-making

Empowering individuals to decide their career paths is essential for a satisfied and productive workforce [23,24]. Increasing volatility in current job markets and other changes in the workplace have relegated the responsibility of career advancement to individuals. Yet, limited resources are often provided to support their career decisions [25]. Accordingly, access to relevant, personalized data (e.g., necessary competencies of desired jobs, training to build competencies, and current and future labor market needs) is essential for career decision-making [26]. Such data enables individuals to establish career goals [27], select training to enhance work-related competencies, and navigate career pathways by identifying opportunities to advance their careers.

#### eMPACT^TM^: addressing stakeholders’ needs

The framework presented in Figure 1 reflects a need for novel solutions to workforce development and guided us to design eMPACT^TM^, a career navigation system, that could promote a cycle of purposeful training and career advancement outcomes. The system collects personal and cumulative data about workplace competencies, local job openings, and training availability from three key stakeholder groups (employees, employers [e.g., Principal Investigators], and training developers/coordinators) and applies them to promote both individual workers’ career advancement and organizational training goals. Each stakeholder group’s needs and contributions are reciprocal and complementary rather than exclusive or contradictory. The full benefits of this system can only be achieved when all three stakeholder groups are committed to using the system. For example, employees need access to available job and training data to make informed career decisions. This information is usually provided by employers and training coordinators. Likewise, employers and training coordinators need data on employees’ current competencies and targeted job or career goals. These data can assist employers in their recruitment efforts by identifying individuals with the desired skills and qualifications for specific positions. Training coordinators can also benefit from these data to develop effective training programs catering to employees’ needs and aspirations.

Details of each stakeholder group’s needs, benefits (i.e., system functions), and contributions are provided in Table 2.

**Table 2.** Three user groups and their primary needs and their potential benefits from and contributions to eMPACT^TM^

User group	Needs	Benefits (System functions)	Contribution
Employee (CRCs)	Purposeful training & career advancement	Navigating tailored training opportunities	Individual’s current competency levels and interested jobs
		Navigating current and future job opportunities	
		Reflecting on personal profiles to develop career plans	
Employer (PI, HR)	Efficient candidate recrument	Identify and recruit qualified candidates	Job information with competencies
		Simulate and forecast HR needs	
Training Coordinator	Needs-based training offer	Identify and recruit trainees	Training information with expected outcomes
		Simulate and forecast training needs	

CRCs = clinical research coordinators; PI = principal investigator; HR = human resources representative.

*Employee’s purposeful training and career advancement*. CRCs initially engage the eMPACT^TM^ career navigation system by completing a brief questionnaire highlighting key work knowledge and skills (e.g., demographic information, task competency levels, and personal attributes). Knowledge about self is a critical first step in the career navigation process [28]. This knowledge is organized into a personal profile that serves as a baseline for identifying future job aspirations and training needed to successfully achieve an individual’s career goals.

*Principal investigators (PIs) and HR for efficient employee recruitment.* Employers (PIs/HR) engage the career navigation system by posting profiles of available jobs, using an intake survey similar to the one used by employees. Posted job information includes required education and experience levels and required or preferred competencies of the position. In addition, potential employers receive aggregated data that summarizes system information about potential, albeit anonymized, candidates (i.e., individuals that match job requirements). Employers can send recruitment letters to matching candidates who opt for this feature. Aggregated data also provides a broader perspective of the local labor pool.

*Training coordinators/developers for needs-based training offers*. Individuals responsible for developing/coordinating organizational training contribute to the system by entering new or future training program profiles that reflect intake survey data. Training program profiles include information about training levels (e.g., entry level, advanced) and expected learning outcomes based on the same competency survey used by CRCs. A training profile generates an aggregated data report on potential trainee needs. Recruitment emails can be disseminated to candidates (albeit, anonymized for those selecting this feature). Training program profiles can identify individuals with compatible training needs and forecast future training needs.

### Phase 2: Developing a Metric to Represent Competencies

To identify the distinct needs of our three stakeholder groups and offer information customized to their different needs, it was essential to have a unified metric to represent the competency levels that individuals possess, job positions required, and training programs addressed. Key features of eMPACT^TM^ can be compared to car navigation systems that calculate the distance between two points and provide various routes based on map data. Similarly, eMPACT^TM^ calculates the gap between an employee’s current work competencies and those needed for a targeted job. This information is used to identify training recommendations that close the gap. To calculate competency gaps and recommend appropriate job or training opportunities, a competency survey instrument consisting of core-task items was developed and used as a unified metric system to represent competency profiles of relevant stakeholders (viz., individuals, jobs, training).

#### Methods

The eight competency domains for Clinical Research Professionals (Joint Task Force for Clinical Trials Competency [JTF] [29]) provided the basis for the classification of the task competency. An exhaustive list of 263 tasks performed or expected by CRCs was generated through an analysis of three data sources: (a) job descriptions posted by three major medical institutions in the Atlanta metro area, (b) interview data with current and former CRCs collected during needs assessment [30], and (c) core competency guidelines for CRCs developed by the Association of Clinical Research Professionals [31]. Two subject-matter experts were asked to sort and assemble 263 task statements associated with CRC duties (on separate index cards, along with blank cards to add missing tasks) into categories. Experts sorted and combined tasks until a consensus was reached. Ultimately, this process reduced the 263 tasks into essential (or core) task statements.

#### Results

A total of 44 task statements were identified through the expert analysis process and then classified into one of JTF’s eight competency domains (see Supplementary Table 1 for the final version of the survey). Six proficiency levels were developed (see Supplementary Table 2) to indicate employee competency on each task statement [32]. The survey reflecting these statements was reviewed by two experts for validity, tested, and refined by two practicing CRCs.

### Phase 3: Task-Profile Matching Algorithms Development

The 44-item competency survey provided a unified metric to represent the competency profile of individual employees, job positions, and training programs. Survey results are used to calculate matching scores for employees about jobs and training. Matching scores can be used for multiple purposes (e.g., recommendations for customized training, available job openings, listing top-matching candidates for a particular job, and forecasts of potential training needs). This section briefly explains the core matching algorithms.

#### Individual and job matching algorithm

Consider the scenario: An individual wants to know how much his or her competency profile matches the skills profile of a particular job. Proficiency levels, provided by individual responses to the 44 competency-task survey, represent one’s current competency profile and can be represented by vector, 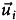 , denoting the individual’s proficiency level in task *i*. Similarly, a job’s required competency profile can be represented by vector, 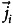 , indicating the job’s required proficiency level in task *i*. To calculate the matching score of an individual to a particular job, a set of relevant tasks (*RT*) for the job (proficiency level in task *i* for the job is not zero) is identified.

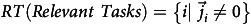




Next, a matching score can be calculated by obtaining the ratio of the sum of a user’s proficiency levels on relevant tasks to the sum of a job’s required or preferred proficiency levels on the same tasks. To avoid overinfluence of a user’s overqualified skills during the matching score calculation, relevant tasks *(i)* are classified into two sets: underqualified tasks, the tasks of a user’s proficiency level that are at or below the job’s required level, and overqualified tasks (OT), the tasks of a user’s proficiency level that are greater than the job’s required level. Then, the influence of OT on the overall matching score is adjusted by adding a coefficient (*ω* = 0.8 ∼ 1) to the following:

*UT*(*Underqualified tasks*) = {*i* ∈ *RT* | 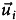 ≤ 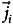 }

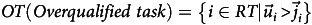




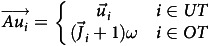




Accordingly, when a user is overqualified in a specific task, *i*, for a job, its influence on the matching score is limited, while underqualified tasks are fully accounted for in the matching score. This allows the system to embrace less matching trainings in its recommendation list to make sure users do not miss any potential training opportunities. For each job, an adjusted vector, 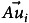 , is calculated to reflect each user’s proficiency level for each task, *i*. Then, a user’s matching score for a job is represented as:

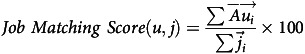




#### Individual and training matching algorithm

Suppose an individual has competency gaps in certain tasks for a particular job. We want to assign a matching score for a certain training (*T*) according to the degree of potential benefits of the training in filling the competency gaps. First, we need to identify a set of tasks where an individual’s competency levels are below the job’s required levels (*TG, Tasks with Gap*), represented as *TG* = {*i*| 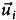 < 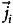 }. Among the tasks with the gaps, we need to check if the individual’s current task levels ( 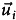 ) for the task are in the range of the training’s target audience levels by checking the training’s entry-level (i.e., the minimum level needed for an effective learning experience) and maximum level of expected learning outcome for each selected task. We denote minimum and maximum levels of a task, *i*, by two vectors, 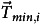 and 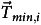 . Of the tasks for which an individual does not meet job requirements (*TG*), we consider two sets:

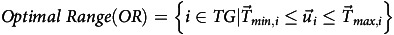




*Not Optimal Range*(*NOR*) = 
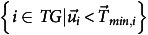


*Optimal Range (OR)* refers to a set of tasks (*TG*) where the individual’s task level is in the optimal range for training’s effectiveness. In contrast, *Not Optimal Range (NOR)* refers to a set of tasks (*TG*) where individual competency levels are below the entry level for training, meaning that the training topic is relevant to the individual but may not be as effective as expected. To obtain the training matching score, we count tasks in the optimal range (*OR*) and not in the optimal range (*NOR*), respectively. To account for the possibility that the individual may receive limited benefits from the trainings that are not in the optimal range (NOR), we use a coefficient, *ω* (0.5 as a default value). Finally, we define the training matching score as

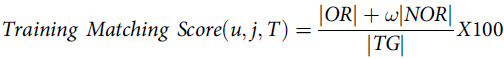




With this algorithm, it is possible to compare different training profiles to each other according to an individual’s competency gap for a job. In other words, the system can recommend highly-tailored training for individuals with their desired jobs.

We further refined the algorithms by differentiating required and preferred tasks for a job. We also considered other qualifications (e.g., degrees, certificates, and years of experience) as filters to screen applicable jobs for an individual before calculating matching scores. Notably, the same algorithms and resulting scores are used to recommend top marching candidates for a job and potential trainees for training to serve organizational stakeholders (e.g., employers and training coordinators).

### Phase 4: Algorithm Validation through Simulation Testing

A simulation test was conducted for algorithm validation. While hypothetical user profiles were used, the real data were used for jobs and training. We used CRC positions available in the Atlanta metro area during the testing period (over 100 real job positions). Similarly, we used free online training courses for clinical research professionals that were developed and offered through the Georgia Clinical and Translational Science Alliances (Georgia CTSA) Course Catalog (https://twd.ce.emorynursingexperience.com/), in collaboration with the University of Southern California Clinical and Translational Science Institute.

#### Methods

Five hypothetical employee profiles were created to represent various levels in the progression of CRC careers, from an entry-level (CRC I) to an advanced level (CRC IV), with corresponding competency levels on the 44 tasks and basic qualifications (see Supplementary Tables 1 and 2). With the profiles, we simulated five scenarios and collected the eMPACT system’s recommendations of jobs and the trainings for pursuing a selected matching job position.

#### Results

**Figure 2.**
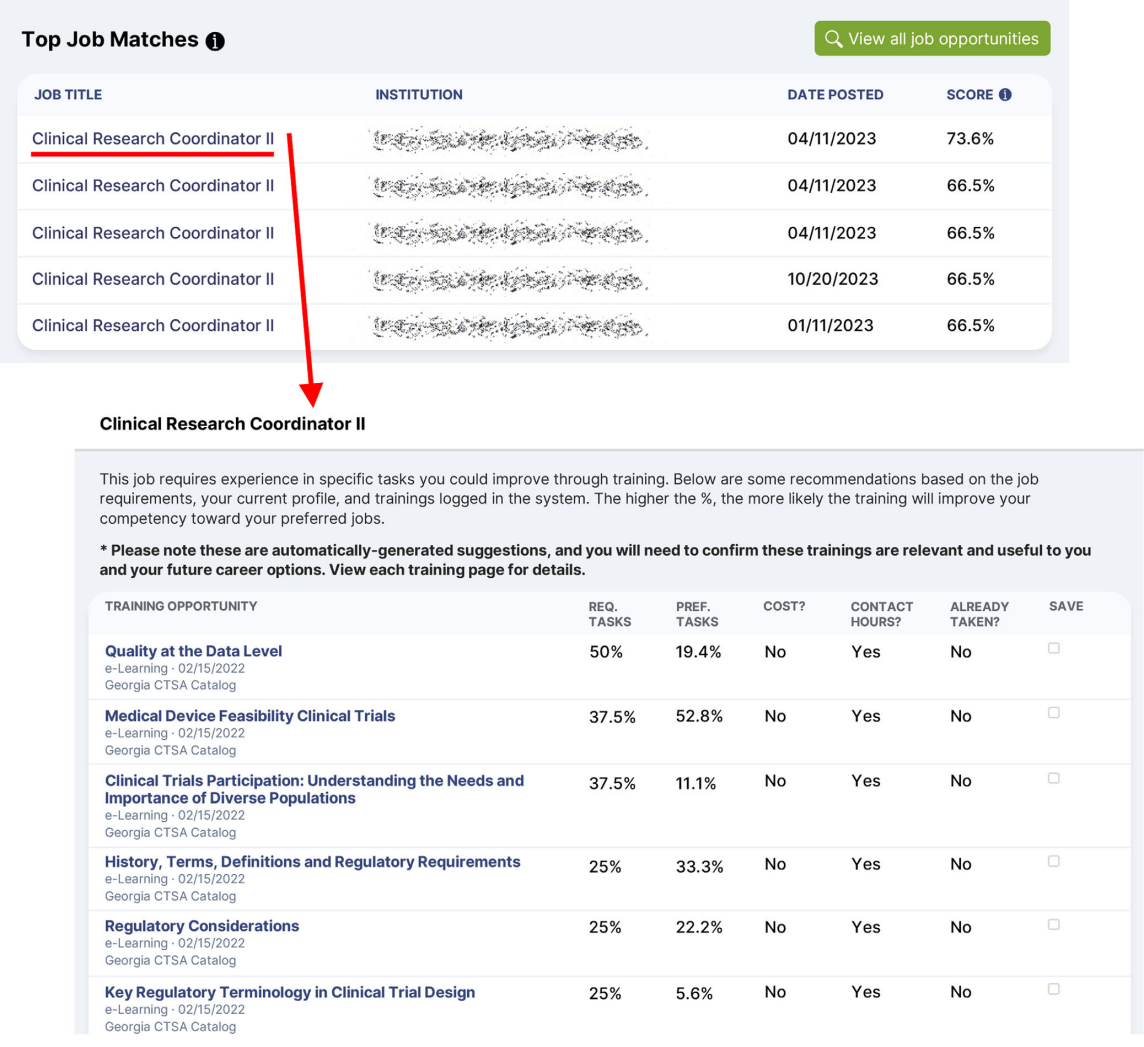
Sample screen captures for top job and training matches of a hypothetical user holding a current position of clinical research coordinator I.

The simulation test results demonstrate eMPACT’s potential in supporting CRCs in their career planning and advancement. Sample screens of eMPACT demonstrating the Case 1 scenario are shown in Figure 2. For a hypothetical CRC I profile with an entry-level competency, the system recommended five CRC II positions through Dashboard, with the top-matching job calculated as a 74% match. The 74% matching score indicates the extent to which the candidate’s competency profile meets the required competency profile of the job position. The system also recommended training courses, such as *Quality at the Data Level* (50%) and *Medical Device Feasibility Clinical Trials* (37.5%), for the job position to close the gap (26%). Training matching scores indicate the maximum percentage of the gap that could be covered by a given training, according to the training’s learning outcome profile. The remaining scenarios and their results are summarized in Table 3. Notably, while Case 1 and Case 2 had the same level of CRC position (CRC I), Case 2’s slightly higher competency level resulted in a higher matching score (95%) for the same job position (CRC 2 Infectious Disease) compared to Case 1. Case 3 represents an individual with educational and experience backgrounds who qualifies for a CRC 3 position but is currently a CRC 2. The system’s recommendations clearly reveal this candidate’s suitability for available CRC 3 positions (97% match). Case 5 presents a scenario of an advanced-level candidate who currently holds a CRC IV position, and the system recommended a Clinical Research Supervisor position with a matching score of 111%. This means that no job positions were available in the eMPACT database that held matching scores ranging from 90 to 100%, resulting in the system producing the next best alternative – positions the candidate may be slightly overqualified for.

**Table 3.** eMPACT results of job and training recommendations in hypothetical scenarios

Case	Current job title	Self-report competency level	Qualifications	Top jobs recommended (% match)	Top trainings recommended for tob job^a^ (% of gap covered)
1	CRC I	1–2	Bachelors 3 years exp	CRC 2 infectious disease (74%)^a^ CRC 2 cardiology (67%) CRC 2 oncology (67%)	Quality at data level (50%) Medical device feasibility clinical trials (38%) History, terms, definitions and regulatory requirements (25%)
2	CRC I	2–3	Bachelors 3 years exp	CRC 2 palliative medicine (95%)^a^ CRC 2 infectious disease (95%) CRC 2 Winship clinical trials (93%)	Medical device feasibility clinical trials (25%) Quality at data level (25%) Regulatory considerations (25%)
3	CRC II	2–4	Bachelors, Masters	CRC 3 infectious disease (97%)^a^	Concerns in global clinical trials (50%)
			10 years exp	CRC 3 cardiology (97%)	FDA initiatives to address diversity in clinical trials (25%)
			ACRP-CP	CRC 3 pediatrics; cystic fibrosis (97%)	Clinical trials transformative initiative approach (25%)
4	CRC III	3–4	Bachelors	CRC 4 oncology (88%)^a^	Concerns in global clinical trials (22%)
			7 years exp	Clinical research navigator (88%)	Regulatory considerations (11%)
			CCRC	Clinical research supervisor (87%)	Clinical trials transformative initiative approach (11%)
5	CRC IV	4–5	Bachelors, Masters	Clinical research supervisor (111%)^a^	N/A
			11 years exp	CRC 4 oncology (111%)	
			CCRP	CRC 4 pulmonology (111%)	

ACRP-CP = association of clinical research professionals-certified professional; CCRC = certified clinical research coordinator; CRC = clinical research coordinator; exp = experience; FDA = The United States food and drug administration; N/A = Not Applicable.

a Indicates the top job recommended for each scenario. The results of training recommendations are based on the top job. The results vary depending on the selected job and user competency profile.

While the dashboard (see Fig. 2) is designed to display a user’s top five matching job positions as well as top five matching training recommendations according to his or her saved target job positions, users also have access to a full list of job opportunities available in the eMPACT database that they can review and filter by critical factors, such as salary or location. They can explore positions at different levels and institutions, gaining insight into the types of competencies required by employers across the spectrum. Should they be interested in pursuing a particular position, immediately or as a next step in their career, they are provided direct links to related trainings that can support them. A full list of training opportunities is also available for review.

The system uses identical algorithms for all three user groups; thus, validation for one group (employee) grants the validation for the other groups (employer and training coordinator/developer).

### Phase 5: System Evaluation through Early Dissemination

The eMPACT service was fully launched in Georgia in mid-October 2022 and promoted through the Georgia CTSA network. During this early dissemination phase (Oct 2022–May 2023), we primarily aimed to recruit CRCs in Georgia because a larger CRC user population is essential to promote other stakeholder groups’ participation such as PIs and training developers. Accordingly, we entered and updated CRC job data in the Atlanta metro area and free online training data relevant to CRC positions offered through the Georgia CTSA Course Catalog. The results of early dissemination to CRCs were reported in this section.

#### Number of visits to eMPACT

The eMPACT website had a total of 2,509 visits during this early dissemination period. Although we targeted CRCs in Georgia (969 visits, 38.6%), there is a significant number of visits from other states and countries worldwide (see Table 4). The average duration of each visit was approximately 3 minutes for new visitors and 5.3 minutes for returning visitors.

**Table 4.** eMPACT site visit geological data (Oct 14, 2022–May 31, 2023)

		*N* = 2,509	
		*n*	*%*
Nationwide (top 10)	Georgia	969	38.6
	Texas	307	12.2
	Virginia	148	5.9
	California	141	5.6
	Iowa	94	3.7
	Florida	86	3.4
	New York	58	2.3
	Illinois	50	2.0
	Ohio	39	1.6
	Unknown	183	7.3
	Other^a^	434	17.4
Worldwide	North America	2,298	91.6
	Asia	109	4.3
	Europe	68	2.7
	Oceania	18	0.7
	Africa	9	0.4
	South America	6	0.2
	Central America	1	0

a Includes foreign countries.

#### eMPACT user demographics and workforce competency data

**Figure 3.**
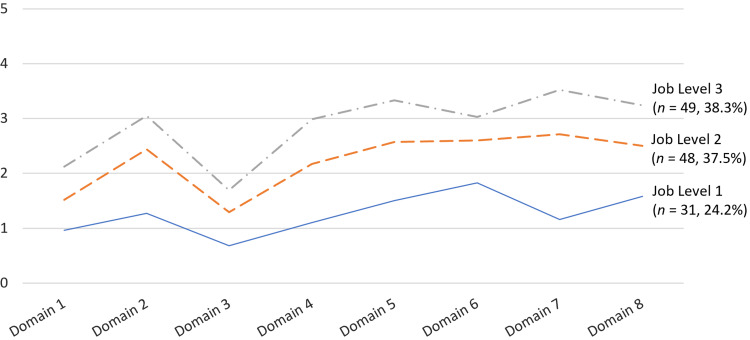
Plots for mean scores of the eMPACT users’ perceived proficiencies on core competency domains for clinical research according to their job levels. *N* = 128. The joint task force for clinical trial competency core competency framework for clinical research professionals [29]; Domain 1, scientific concepts and research design; Domain 2, ethical and participant safety considerations; Domain 3, investigational products development and regulation; Domain 4, clinical trial operations; Domain 5, study and site management; Domain 6, data management and informatics; Domain 7, leadership and professionalism; and Domain 8, communication and teamwork; 6-point Likert-type response scale (0, no experience/not applicable; 1, basic understanding; 2, perform with supervision; 3, perform independently; 4, take initiative and train others; and 5, recognized authority); job level 1, handling basic administrative duties related to clinical trials; job level 2, handling key administrative and monitoring duties related to clinical trials; and job level 3, independently leading, managing, and providing expertise across all areas of study operations.

A total of 192 CRCs (employees) were registered users of the eMPACT^TM^ system as of May 31, 2023. Their demographic statistics showed diverse backgrounds in gender, age, ethnicity, and job level (see Table 5). The educational level of most users was at or above a bachelor’s degree. The system also captured workforce competency data according to the user’s job levels (see Fig. 3). The CRC workforce reported a relatively lower level of perceived competency in Domain 3, investigational products development and regulation, and Domain 1 – scientific concepts and research design – while they perceived relatively higher competency in Domain 5 – study and site management. A similar competency pattern among CRCs was also found in a recent study [33].

**Table 5.** Demographics of eMPACT users overall (Oct 14, 2022–May 31, 2023)

		*N*	*%*
Gender	Woman	126	82.9
	Man	22	14.5
	Prefer not to answer	4	2.6
	Total	156	100.0
Age	20s	39	20.4
	30s	68	35.6
	40s	42	22.0
	50s	33	17.3
	60s	8	4.2
	70s	1	0.5
	Total	191	100.0
Race/ethnicity	African American	56	33.1
	Asian	22	13.0
	Latino/a	9	5.3
	White	58	34.3
	Some other race	6	3.6
	Prefer not to answer	18	10.7
	Total	169	100.0
Education	High school diploma or GED	2	1.0
	Some College	6	3.1
	Technical Degree/Certificate	3	1.6
	Associate Degree	4	2.1
	Bachelor’s Degree	70	36.6
	Master’s Degree	67	35.1
	Doctor of Philosophy	11	5.8
	Medical Degree	7	3.7
	Bachelor of Medicine, Bachelor of Surgery	7	3.7
	Doctor of Pharmacy	1	0.5
	Total	178	100.0
Job Level ^a^	Level 1	31	24.2
	Level 2	48	37.5
	Level 3	49	38.3
	Total	128	100.0

GED = general educational diploma.

a Each institution may use different job-level systems. A generic three-level system was developed to accommodate institutional differences. Level 1 indicates CRC job positions handling basic administrative duties related to clinical trials. Level 2 indicates CRC job positions handling key administrative and monitoring duties related to clinical trials. Level 3 assumes independently leading, managing, and providing expertise across all areas of study operations. Users selected a corresponding job level according to their job duties.

Overall, the growth pattern reflected in site visits by registered users was steady, while their demographic characteristics were diverse. The workforce statistics captured by the system offered the characteristics of the CRCs in Georgia, manifesting the potential to support informed decisions on workforce development at an individual, organizational, and state level.

## Discussion and Future Directions

Recruiting, educating, and maintaining a qualified workforce is essential for advancing translational science and healthcare practice. As an alternative to organization-driven training, we envisioned a pragmatic workforce development approach serving both individual and organizational needs. CRCs were selected as our initial focus since they represent a large sector of clinical research professionals and experience a relatively high turnover rate [8]. We conceptualized a career advancement cycle with purposeful training first. Then we developed, tested, and implemented a career navigation system, eMPACT, that supports CRC career advancement while concurrently addressing the organizational training needs of employers and training coordinators. Although the project’s five most representative phases and critical outcomes were presented linearly, this project involved multiple iterations and refinement over time. The results of the early dissemination of eMPACT show its potential to help understand the CRC workforce and support its diversity. This section further discusses our underlying pragmatic workforce development approach, the accuracy and benefits of self-appraised competency profiles, and the need for long-term impact analysis, suggesting implications for future research and development.

### A Pragmatic Workforce Development Approach

Despite the prevailing training paradigm focusing on organization-driven workforce development [11], we believe that an individual-centered perspective toward workforce development and training can contribute to the success of both individuals and organizations [34]. This perspective posits that supporting employee efforts to establish and attain their professional career goals will result in greater job satisfaction and, in turn, lead to a more productive and healthy organization. To actualize this perspective, we considered a mutual database platform (eMPACT^TM^) that facilitates individual workers to navigate and advance their careers, but that does not eliminate organization-centered training efforts. Rather, we envisioned that implementing an individual-centered career support database system could reflect an organizational commitment to empowering individual workers by providing opportunities to acquire knowledge of self, training opportunities, and employment (labor market) information. This broader, pragmatic approach recognizes the value inherent in both worker- and organization-driven workforce training perspectives.

We further believe that integrating individual- and organization-centered approaches can truly promote the roles and goals of all stakeholders. An individual-centered approach is based on personal agency and support in acquiring information about self (e.g., knowledge of professional abilities and career goals) and customized information about training opportunities and the local labor market (employment opportunities and requirements). This integrated approach recognizes the mutual responsibilities and benefits shared by key stakeholders in workforce development. As a result, training becomes a more meaningful and strategic effort for individuals and organizations. Organizations have richer information about professional development needs allowing for purposeful and targeted, rather than haphazard, approaches. Ultimately, individuals can determine desired professional goals, understand work requirements for career advancement, and identify gaps between current competencies and needs. In addition, organizations benefit from a better understanding of existing worker characteristics, expressed training needs, and available work opportunities.

Our efforts for developing and disseminating a mutually beneficial database platform, such as eMPACT, in one sector of the clinical research workforce, such as CRCs, could also be seen as an effort to validate our pragmatic workforce development approach.

### Self-Appraisal of Competencies: Accuracy and Benefits

Valid individual competency profiles are essential for the success of our career navigation system as they contribute to (a) identifying and securing professional development (training) and (b) integrating self-knowledge into a comprehensive career plan [7]. We selected the use of self-appraisal rather than standardized assessments. While self-appraisal has limits, the role of assessing and developing one’s own professional competence is important in both initial and ongoing job preparation and development of health professionals [35,36]. Self-assessment is also an important influence on self-regulation and the ability to reflect on professional practice [37]. Notably, a self-appraisal approach aligns with our fundamental commitment to empowering individual users in the career navigation process. Well-guided self-appraisal can offer a more holistic and proactive self-reflection experience [38,39] than standardized assessment. Standardized assessments are often expensive and difficult to design and maintain valid and reliable test items. Undoubtedly, the dynamic nature of CRCs’ roles would further contribute to the costs of time and resources.

To increase the accuracy of self-appraisal of professional competency, the system promotes awareness of the benefits of accurate self-appraisal – more precise self-appraisal results in more meaningful and relevant jobs and training recommendations. In addition, real-time changes in recommended job and training options are available as individuals adjust their profiles. This process can help understand and reflect on competencies. Future research is warranted on the key factors and strategies that influence and improve the accuracy of self-appraisal data.

Training coordinators may encounter challenges in representing existing training programs using the 44-item competency survey developed for CRCs, especially since few trainings were designed to specify information such as targeted competency domains and tasks, or specific learning outcomes tied to the system. Future efforts should seek to facilitate training developers’ ability to identify targeted competencies and tasks, during the early stages of developing new training programs. These efforts will increase the accuracy of training profile data and the quality of training.

In a similar vein, future efforts are planned to provide employers with more accurate and efficient support in the recruitment of matching candidates, such as clearly defining job requirements using the same competency profile. This coordinated process allows employers to articulate the roles and boundaries of a position, which could lead to better communication with future employees.

### Long-Term Impact On Sustainable Translational Workforce Development

Research is needed on the long-term impact of eMPACT service on CRC workforce development and organizational training goals to advance theory and practice. In particular, long-term changes in employees’ career advancement and diversity, job satisfaction, training satisfaction, turnover rate, and burnout will be investigated. Similarly, the benefits of the system for employers (e.g., candidate recruitment) and training developers (e.g., effective training development, targeted learner recruitment) should also be gauged.

These long-term research efforts will leverage and validate our pragmatic approach to workforce development. Our paradigm integrates individual and organizational goals in a coordinated process that empowers individual workers to manage their career paths effectively while ensuring that organizational workforce development needs are achieved efficiently. Our growing understanding of this process and subsequent theoretical and practical advancements will expand our innovations to incorporate other CRP sectors and beyond.

The sustainable growth and long-term benefits of eMPACT users require maintaining up-to-date data on CRC user profiles, training programs, and job positions. We plan to recruit PIs and hiring managers as eMPACT users who can provide and update their new CRC job positions. Similarly, we also plan to develop partnerships with other CTSA program institutions that can provide information about qualified training programs. The information providers of either training or job opportunities will, in turn, be able to access opt-in matching candidates. In this regard, eMPACT would be a job searching or candidate recruitment tool. However, the primary purpose of eMPACT is to provide CRCs with career information, such as training and job opportunities, to build their short- and long-term career goals and access tailored training opportunities to achieve them. Empowering CRCs to access career information and reflect and make decisions on their career will enhance CRCs’ career identity and professionalization for sustained growth of the CRC workforce [40].

## Supporting information

Choi et al. supplementary material 1 Choi et al. supplementary material

Choi et al. supplementary material 2 Choi et al. supplementary material

## Supplementary material

The supplementary material for this article can be found at https://doi.org/10.1017/cts.2023.693.
