# Supplementary material for: Conceptualization, development, and early dissemination of eMPACTTM: A competency-based career navigation system for translational research professionals
Source: J Clin Transl Sci. 2023 Dec 11;8(1):e2. doi: 10.1017/cts.2023.693 (PMC10879852; doi:10.1017/cts.2023.693)
Supplement: Choi et al. supplementary material 1 — Choi et al. supplementary material [file S2059866123006933sup001.docx]

**Supplementary Table 1.** *The final version of the 44-task competency survey*

| **Domain** | **Task Competency** |
| --- | --- |
| Scientific Concepts & Research Design | - Research grant applications |
|  | - Completing a literature review to justify study background |
|  | - Developing study designs |
|  | - Developing study budgets |
| Ethical and Participant Safety Considerations | - Managing ethical and professional conflicts |
|  | - Managing clinical study fraud and misconduct |
|  | - Recognizing, reporting, and following-up on adverse events |
|  | - Institutional Review Board (IRB) preparation, submission, and modifications |
|  | - Institutional Review Board (IRB) reporting and termination |
| Investigational Products Development and Regulation | - Pharmacovigilance |
|  | - Investigational product use instruction |
| Clinical Trial Operations (GCPs) | - Monitoring compliance |
|  | - Resolving issues related to study protocols |
|  | - Clinical audits and inspections |
|  | - Preparing regulatory documents |
|  | - Tracking regulatory submissions |
| Study and Site Management | - Study protocol, feasibility, and initiation |
|  | - Completing essential documents |
|  | - Completing/managing study close-out |
|  | - Study monitoring according to protocol |
|  | - Administrative activities associated with clinical trials |
|  | - Educating research staff |
|  | - Managing the pre-award grant process |
|  | - Managing the post-award grant process |
|  | - Order and maintain equipment and supplies |
|  | - Collection, processing, and shipping of samples |
|  | - Complete the start-up process with study subjects |
|  | - Enrolling, consenting, and educating study subjects |
|  | - Preparing, implementing, and monitoring data collection |
|  | - Managing close-out process with study participants |
| Data Management and Informatics | - Reviewing and report a medical record |
|  | - Collection of data with Electronic Data Capture (EDC) system/Case report form |
|  | - Data analysis |
|  | - Managing research databases |
|  | - Ensuring security of data |
| Leadership and Professionalism | - Supervisory duties |
|  | - Guiding and instructing my staff to provide quality experiences |
| Communication and Teamwork | - Interpreting results and making recommendations for improvement |
|  | - Presenting and communicating research findings to a professional audience |
|  | - Producing scientific publications/publishing research results |
|  | - Interprofessional collaboration |
|  | - Organizing study meetings |
|  | - Leading meetings |
|  | - Communicating with sponsors and/or key business partners |
