# Supplementary material for: Conceptualization, development, and early dissemination of eMPACTTM: A competency-based career navigation system for translational research professionals
Source: J Clin Transl Sci. 2023 Dec 11;8(1):e2. doi: 10.1017/cts.2023.693 (PMC10879852; doi:10.1017/cts.2023.693)
Supplement: Choi et al. supplementary material 2 — Choi et al. supplementary material [file S2059866123006933sup002.docx]

**Supplementary Table 2.** *Proficiency scale for assessing professional competency levels of the 44 tasks*

| Likert-type scale | Proficiency level | Definition |
| --- | --- | --- |
| 0 | Not applicable | Not applicable for job or no relevant experience |
| 1 | Basic understanding | Some knowledge of basic techniques/concepts |
| 2 | Perform with supervision | Limited experience; requires help with tasks |
| 3 | Perform independently | Able to successfully complete tasks. Help occasionally required, but skills usually performed independently |
| 4 | Take initiative and train others | Able to perform tasks without assistance. Recognized within the organization as “a person to ask” when difficult questions arise |
| 5 | Recognized authority | Recognized authority on skills/tasks. Routinely provides guidance, troubleshoots, and answer questions |

*Note*. The proficiency levels presented in this table were developed by adapting the NIH Proficiency Scale (National Institutes of Health, n.d.)
